# Fine Motor Skills and Lexical Processing in Children and Adults

**DOI:** 10.3389/fpsyg.2021.666200

**Published:** 2021-05-12

**Authors:** Rebecca E. Winter, Heidrun Stoeger, Sebastian P. Suggate

**Affiliations:** Department of Educational Sciences, University of Regensburg, Regensburg, Germany

**Keywords:** fine motor skills, lexical development, vocabulary, body-object interaction, body-part association

## Abstract

Children’s fine motor skills (FMS) link to cognitive development, however, research on their involvement in language processing, also with adults, is scarce. Lexical items are processed differently depending on the degree of sensorimotor information inherent in the words’ meanings, such as whether these imply a body-object interaction (BOI) or a body-part association (i.e., hand, arm, mouth, foot). Accordingly, three studies examined whether lexical processing was affected by FMS, BOIness, and body-part associations in children (study 1, *n* = 77) and adults (study 2, *n* = 80; study 3, *n* = 71). Analyses showed a differential link between FMS and lexical processing as a function of age. Whereas response latencies indicated that children’s FMS were associated with “hand” words, adults’ FMS linked to the broader concept of BOI. Findings have implications for shared activation theories positing that FMS support lexical processing.

## Introduction

Language development is perhaps the most anticipated event in the early years, providing the key to communication, cognitive development, and academic success (Catts et al., 2002). Somewhat counter-intuitively, fine motor skills (FMS) have shown links to vocabulary development (e.g., Cameron et al., 2012; Suggate and Stoeger, 2014, 2017). Current understanding on embodied cognition posits that lexical representations contain sensorimotor information (e.g., Pexman, 2019). However, little work has examined whether actual performance in the sensorimotor system, and therewith FMS, relates to lexical knowledge. Thus, research as to why two seemingly unconnected constructs relate, namely FMS and vocabulary, is still sparse and inconclusive, requiring more study and theoretical development. In the current study, we seek to replicate and extend previous work by examining the role that FMS (Suggate and Stoeger, 2014, 2017) play in lexical processing in both adults and children. Additionally, we seek to test for unique roles attributable to sensorimotor information, via the extent to which words invoke body-object interactions (Siakaluk et al., 2008a,b) and body-part associations (James and Maouene, 2009).

### Links Between Fine Motor Skills and Vocabulary Development

FMS can be described as the use of small muscles in movements involving the (manual) extremities when, for example, manipulating objects (Gaul and Issartel, 2016). FMS often involve close hand-eye coordination, such as when writing, turning pages or using a computer keyboard. Theories on links between FMS and vocabulary posit three main explanations. First, a general developmental factor (maturation) has been suggested as the driving force (e.g., Gesell and Thompson, 1934), whereby development in FMS runs parallel to development in language, leading to spurious non-causal correlations. However, subsequent studies have shown that maturation cannot explain the link entirely (e.g., Cameron et al., 2012; Suggate and Stoeger, 2014, 2017).

A second account (functionalism) proposes, that FMS contribute actively to the development of vocabulary, in the sense that children with better motor skills have greater interactions with the environment, which introduces possibilities to explore and expand their lexicons (Iverson, 2010).

Thirdly, the shared activation hypothesis suggests that FMS and vocabulary utilize similar underlying brain structures and processes, such as the Broca-area, the cerebellum, or the pre-frontal cortex (e.g., Glenberg and Kaschak, 2002; Pulvermüller, 2005; Anderson, 2007). Presumably, processing of words related to FMS might involve an inner re-experiencing of those concepts, thus activating the same processes as FMS do (Martzog and Suggate, 2019). An extension to the shared activation account, the nimble-hands, nimble-minds hypothesis (NHNM), suggests that processing skills associated with FMS may be used for processing embodied lexical items (Suggate and Stoeger, 2014, 2017).

To date a significant amount of evidence has linked FMS and vocabulary development (word comprehension). In a large-scale study investigating children’s academic achievement and FMS, robust links between FMS and receptive vocabulary from kindergarten to second grade were found (Pagani et al., 2010). Cameron et al. (2012) assessed the contribution of FMS to academic achievement in kindergartners and found a relationship between FMS and expressive vocabulary (Woodcock, 1998). Similar results were reported by Dellatolas et al. (2003), in that the degree of FMS related strongly to a vocabulary measure comprised of receptive and expressive vocabulary tasks (picture naming) in children aged three to six. Furthermore, children on the autism spectrum disorder with poor language abilities show large deficits in manual dexterity (Dziuk et al., 2007). Taking very early FMS development into consideration, a growing body of research shows a clear link between children’s object exploration and language skills, such as vocabulary size (Ruddy and Bornstein, 1982) or word comprehension (Sansavini et al., 2010).

However, studies also show contradicting results. Wassenberg et al. (2005) assessed cognitive and general motor performance in a sample of 378 children aged five to six years. Minimal evidence for links between general motor performance and cognitive skill was found and a visuo-motor integration task involving copying forms, which bears similarities to FMS through requiring precise pencil operation, did not predict vocabulary. A study by Alcock and Krawczyk (2010) found that although parent-reported FMS and language development related closely, no association existed on a standardized developmental assessment scale for motor skills (Bayley Scales of Infant Development, Bayley, 2006) and language skills in children.

In summary, these mixed findings generally point to a role of FMS in vocabulary development, although findings are somewhat inconclusive and limited to children and little is little known about underlying mechanisms. As discussed next, research on lexical processing has identified sensorimotor word features that are associated with faster response latencies, which may provide an avenue to better understand links between FMS and vocabulary.

### Body-Object Interaction

Research suggests that lexical items are not stored exclusively in amodal information structures, but instead in complex networks involving the sensorimotor system (Glenberg and Gallese, 2012; Pexman, 2019; Kiefer and Harpaintner, 2020). Neuropsychological studies indicate that sensorimotor cortices are activated when adult participants hear words pertaining to action words (Hauk et al., 2001; Pulvermüller et al., 2001) and transcranial magnetic stimulation can interfere with lexical processing (Buccino et al., 2005; Papeo et al., 2009). Moreover, several studies have shown that motor actions and action words share common cortical representations (e.g., Boulenger et al., 2006). Turning to behavioural studies, words are processed differently depending on their degree of sensorimotor information (Siakaluk et al., 2008a,b). Thus, generally, abstract words are processed more slowly than concrete ones (Maouene et al., 2011; Amsel and Cree, 2013) and processing advantages exist for lexical items that are perceptually rich (Connell and Lynott, 2012; Hargreaves et al., 2012a).

One index for the degree of sensorimotor information relates to the degree of body-object interaction (BOI), referring to the “extent to which a human body can physically interact with a word’s referent” (Pexman, 2012, p. 12). High-BOI words include those rated as easy to interact with, such as *chair* or *couch*, and the opposite applies to low-BOI words (e.g., *sky*, *star*). In a series of experimental studies, Siakaluk et al. (2008a, b) tested for a BOI effect on visual word recognition and semantic processing in undergraduate university students. Stimuli were matched on imageability, concreteness and other lexical and semantic variables known to have an influence on processing speed. In both tasks, facilitatory effects were found for the high-BOI words with participants reacting more quickly and accurately to high- versus low-BOI words. This pattern of results has been observed in adults for nouns and verbs (Tillotson et al., 2008; Bennett et al., 2011) as well as in different experimental settings using manual tasks (e.g., button press responses; Siakaluk et al., 2008b) and non-manual tasks (e.g., verbal responses; Wellsby et al., 2011). Further, neural correlates have been found for high-BOI words (Hargreaves et al., 2012b).

Although studies with adult participants have shown robust processing advantages for high-BOI vocabulary, those with younger populations yield mixed results. A study by Wellsby and Pexman (2014) assessed the development of the BOI effect in children, aged six to nine years, and adults. A facilitatory BOI effect was only found in older children (aged 8 and 9) and adults, but not in younger children (aged 6 and 7). However, as the task demanded a high degree of reading ability, it may not have been suitable for children of that age group. In a subsequent study by Inkster et al. (2016), the authors used an auditory lexical decision task, finding a strong facilitatory BOI effect in six to seven year-old children.

### BOI, Body-Part, and FMS

Given lexical processing advantages for words with a high-BOI rating, a question arises as to whether such lexical items especially relate to FMS at a behavioural level, that is, beyond evidence indicating shared neurological activation. According to the shared-activation and nimble-hands, nimble minds hypotheses, FMS might share neurobehavioral processes with lexical processing, particularly for words that are high-BOI (Suggate and Stoeger, 2017) or related to the hand (Suggate and Stoeger, 2014). Thus, Suggate and Stoeger (2014) tested whether FMS showed a stronger link to high-BOI vocabulary than to general vocabulary. Findings indicated that children’s FMS indeed predicted the processing advantage for high BOI words, even after controlling for age, general vocabulary and other lexical variables. Additionally, they tested whether the extent to which words’ referents could be manipulated by the hand related to FMS, which it did. In two further studies, Suggate and Stoeger (2017) found unique links between children’s response latencies for high-BOI words and FMS, even after controlling for low BOI words, general vocabulary, reasoning and chronological age. However, in a study looking at response latencies for emotionally positive, neutral, and negative words in a samples of five, six, and seven year-olds, no effect of BOI, FMS, or their interaction was found (Lund et al., 2019) – possibly because the words were selected as a function of emotional valence, not BOI.

Alternatively, it may not be essential that the object *per se* is manipulable, but instead which body-part it is typically manipulated with. This assumption is partly supported by a study from Maouene et al. (2008), which found that verbs learned prior to three years of age had a strong association to specific body-parts, namely the hand, foot, eye, or mouth. Additionally, they found that the age of acquisition of the verb related to the associated body-part. In a subsequent fMRI investigation of four-to-six-year-olds, James and Maouene (2009) found that listening to verbs that refer to an action carried out by a certain body-part (leg verbs vs. hand verbs) activates regions in the motor cortex involved in real hand and leg movements. More precisely, verbs associated with hand movements activated different frontal cortex regions than verbs associated with leg movements. These results suggest a role of body-part-associations in the processing of verbs, even at a very young age.

Relating body-part association to the BOI effect, Heard et al. (2018) proposed that certain aspects of motor experience contribute to the BOI effect. Using a sample of 621 words, among different motor dimensions (e.g., graspability, ease of pantomime and number of actions), they found graspability to be a significant predictor of the BOI ratings as well as one of the most significant predictors of semantic processing in an adult sample. This is of particular interest because BOI refers to interactions using any part of the body, whereas graspability focuses primarily on object interactions using hands and fingers. However, Suggate and Stoeger (2014) did not find that words rated as directly manipulable with the hand (e.g., *feather, picking*) showed a greater link to FMS than BOI words. Thus, evidence is mixed as to whether a body-part localized action, or BOI *per se*, or both, mediate links between FMS and vocabulary.

### Current Experiments

To date, research has found mixed evidence of links between FMS and general vocabulary (e.g., Wassenberg et al., 2005; Alcock and Krawczyk, 2010; Grissmer et al., 2010; Pagani et al., 2010; Cameron et al., 2012). According to a shared-activation account, FMS might be used during processing of high-BOI words because this involves an internal simulation of the fine-motor actions associated with the lexical concepts. Two studies have shown that BOI does indeed relate to FMS in children (Suggate and Stoeger, 2014, 2017), providing initial support for the shared-activation account. Additionally, given that BOI is a coarse construct (Lund et al., 2019), and that research finds specific links between body-parts and associated lexical development (Maouene et al., 2008; Hills et al., 2009), it would appear promising to test whether links between lexical development and FMS are governed by body-part association or BOI ratings. However, only one study has looked at the hands and FMS (Suggate and Stoeger, 2014) and needs to be extended to other limbs (i.e., arms, mouth, foot). Indeed, research has found indications that the mouth, hand, and leg might constitute different psycholinguistic systems with different effects on language processing (Ghio et al., 2013; Villani et al., 2019). Finally, previous work has included only children, whereas it is conceivable that BOI and body-part association influences change with age as language becomes more abstract and internalized.

Therefore, in three experiments, we measured FMS as well as BOI and body-part association ratings of select lexical items, alongside a host of lexical and cognitive control variables. Extending previous work (Maouene et al., 2008; Suggate and Stoeger, 2014, 2017; Heard et al., 2018), we examined the contribution of FMS for words associated with the body-parts “hand,” “arm,” “foot” and “mouth.” Our aims were to test whether (a) links between FMS and vocabulary depend on BOI rating, (b) body-part associations explain more variance than BOI, and (c) relations between BOI and FMS transfer to adult samples, as predicted by the shared-activation and nimble-hands, nimble-minds hypotheses.

In experiment 1, we investigated the role of body-part associations in children’s lexical performance (response latencies in a word-picture recognition task), in comparison to BOI while controlling for a host of lexical and cognitive variables. In study 2, we explored whether the results extended to an adult population. Study 3 sought to replicate the findings of study 2, using a different measure of FMS with a higher ceiling to that employed in study 2.

## Experiment 1

To date, only three studies have assessed the relationship between children’s FMS and vocabulary while taking BOI into consideration (Suggate and Stoeger, 2014, 2017; Lund et al., 2019). Two of these studies found that FMS predicted a processing advantage for high BOI words, even after controlling for age, general vocabulary and other lexical variables, whereas the third study did not, possibly due to the focus on emotional valence (Lund et al., 2019). In the current study, we expand the study design by incorporating body-part associations. Given that different studies have shown that children’s vocabulary learning shows a strong connection to corresponding body-parts although not often looking at FMS, this avenue would appear promising. Additionally, we sought to address one methodological limitation in the studies by Suggate and Stoeger (2014, 2017), which used experimenter-operated stop-watches to measure response latencies. Although simple and economic, this method may have lacked accuracy. Therefore, we developed a computerized picture recognition reaction time task. Furthermore, we included different lexical variables, namely: estimated age when children typically acquire lexical items (age of acquisition); the number of related words in the language (semantic neighbourhood); distinctness to other words (semantic diversity), word length, number of syllables, and frequency with which the word is used in the language to ensure that any effect found in the study could either be attributed to the degree of BOI or the body part association. As with Suggate and Stoeger (2014, 2017), children were aged three to six years, the age range found in German preschools, and we controlled for chronological age, and receptive vocabulary, and included an IQ proxy.

First, we expected children to show shorter response latencies to high BOI words, compared to low BOI words, thereby replicating the results by Siakaluk et al. (2008a, b) with a younger age group. Second, in accordance with studies by Suggate and Stoeger (2014, 2017), we expected children’s FMS to predict the response latencies for BOI-vocabulary, in that children with greater FMS show faster lexical processing of high-BOI words. Thirdly, taking the studies by James and Maouene (2009), Maouene et al. (2008) as well as Pulvermüller et al. (2005) into consideration, we tested the additional hypothesis that words associated with the body-part hand show an even greater association with FMS than more general BOI-vocabulary.

### Materials and Methods for Experiment 1

**Participants.** A total of 77 children attending pre-school aged three to six years (*M* = 4.55, *SD* = 0.88) took part in this study, of which 52% were female. A post-hoc power analysis revealed that with four predictors (i.e., age, working memory, vocabulary, and FMS) and the current sample size, assuming a conservative total variance estimate (i.e., *R*^2^ = 0.15), an adequate power of.83 would be obtained. The two pre-schools were located in and around a small city in southern Germany with a population of approximately 160,000 people. Overall, 78% of children were right-handed, 17% left-handed and 5% ambidextrous. On average, 23% of children spoke an additional language to German at home. Regarding educational background, 36% of mothers and 51% of fathers had acquired a university degree. Therefore, this sample was better educated than the average German norm of 28% (OECD, 2016).

**Procedure.** Flyers including information about the study, as well as consent forms and questionnaires, were sent to the children’s parents in advance. Children in the kindergarten whose parents had given their consent (∼50%), were tested individually in their pre-schools. To minimize potential concentration issues, the experiment was divided into two sessions of approximately 30 min. Session one contained the Matrices test, the BOI vocabulary task and the FMS tasks. To establish the dominant hand for the FMS tasks, a handedness test was administered at the beginning of session one by asking children to show how they brushed their teeth and held a pen or pencil. Invariably, children used the same hand to imitate brushing their teeth and writing, which was then recorded as their dominant hand. This was followed by the matrices test. Subsequently, to maintain children’s interest as well as avoid order of sequence effects, the BOI-vocabulary task and the FMS tasks were executed in alteration. Session two included the PPVT, conducted in two blocks separated by the general knowledge task. Half of the children started with Session one, the other half with session two. Sessions were held approximately one week apart. For each finished task the children received a sticker as a reward. The experiment was run by the first-author and trained undergraduate education students.

**Measures.** Measures included demographics, FMS, general vocabulary and reasoning. Demographic information, such as age, gender, spoken languages at home, number of siblings and highest educational qualification of both parents was obtained via a parent questionnaire.

***Fine Motor Skills.*** FMS was assessed with the Movement Assessment Battery for Children (M-ABC 2; Petermann et al., 2011). Tasks entailed posting plastic coins into a slot using firstly the dominant and then the non-dominant hand, threading beads onto a string using both hands simultaneously, and maze tracing. For each task, two trials were possible, depending on whether the first trial was completed within a certain time frame (coins and beads) or without a mistake (tracing). The raw scores represented the number of seconds needed to complete the task (coins and beads) and the number of mistakes (tracing). In accordance with the test manual, the raw scores were converted into normed standard scores.

***BOI Vocabulary Test.*** We adapted a BOI-vocabulary task (Suggate and Stoeger, 2017) suitable for children not yet able to read. Out of the 123 items for the BOI-test, 107 Stimuli were drawn from the Tillotson et al. (2008) database, which contains BOI ratings for English nouns, supplemented by items previously used (Siakaluk et al., 2008a,b; Hargreaves et al., 2012a; Tousignant and Pexman, 2012; Wellsby and Pexman, 2014), and 16 items selected by the authors, creating a pool of 123 items to be translated into German (see Appendix A). To cross-validate the BOI ratings of the English original and the German translations, five randomly chosen department employees rated each of the 123 items on a 7-point Likert scale (*1* = low to *7* = high) as to “…the ease with which a human body can physically interact with a word’s referent” (Siakaluk et al., 2008a). For the German rating, a translated version of the Siakaluk et al. (2008a) instructions was used. Results showed an excellent interrater reliability amongst the five raters, Cronbach’s alpha = 0.93, ICC = 0.93, and again between the raters and the Siakaluk databank, Cronbach’s alpha = 0.87, ICC = 0.87. The average of all five raters was taken as the BOI rating for each item.

Additionally, body-part associations were rated on a 7-point Likert scale (1 = not at all to 7 = very much) based on the question “To what extent do you associate these words with following body-parts?”. Twenty-one different undergraduate university students, who worked in the department as research assistants or were completing undergraduate research projects, rated each word’s association with the hand, arm, foot and mouth. Here again, an excellent interrater reliability was attained, Cronbach’s alpha = 0.96, ICC = 0.96.

To ensure that any effects found in this study could either be attributed to the degree of BOI or to the body-part association, we additionally incorporated different linguistic variables including: age of acquisition, semantic neighbourhood, semantic diversity, length, number of syllables and frequency of the words. We collected the ratings using databases from various studies, such as Bayley’s (2006) for age of acquisition, Durda and Buchanan’s (2006) for semantic neighbourhood, Hoffman et al.’s (2013) for semantic diversity, and the public database Leipzig Corpora Collection^1^ for length, syllables, and frequency. Means and standard deviations for the words ratings (BOI and body-part) and different word variables, can be found in Table 1.

**TABLE 1:** Means, standard deviations, and correlation coefficients for scores on the word ratings (BOI and body-part ratings), picture features (complexity and fit) and word variables (semantic neighbourhood, semantic diversity, length, number of syllables, frequency class).

		*M*	*SD*	1	2	3	4	5	6	7	8	9	10	11	12	13
1	BOI	4.03	1.96	–	−0.19*	−0.17*	−0.09*	0.11*	0.24*	0.41*	0.69*	0.47*	0.03	0.16*	0.40*	−0.25*
2	Age of acquisition	4.50	1.22		–	0.01	−0.15*	0.20*	0.18*	0.30*	−0.02*	−0.00	0.04*	−0.18*	−0.15	0.19*
3	Semantic neighbourhood	10.54	52.53			–	−0.36*	0.04*	0.00	−0.08*	−0.16*	−0.14*	0.10*	−0.01	0.00	0.20*
4	Semantic diversity	1.56	0.20				–	−0.08*	−0.13*	−0.28*	−0.02*	0.03*	0.11*	−0.21*	0.03	−0.06
5	Length	5.50	1.93					–	0.70*	0.40*	0.18*	0.14*	−0.16*	0.06*	−0.11	0.12
6	Number of syllables	1.67	0.06						–	0.43*	0.19*	0.15*	−0.00	0.11*	−0.04	0.06
7	Frequency class	11.58	2.11							–	0.28	0.21	−0.04	−0.05	0.03	−0.06
8	Hand rating	3.67	1.39								–	0.65*	0.10*	0.13*	0.16*	−0.10
9	Arm rating	2.71	1.01									–	0.28*	−0.21*	0.07	−0.05
10	Foot rating	2.35	1.48										–	−0.04*	−0.08	−0.08
11	Mouth rating	2.53	1.87											–	-11	−0.01
12	Picture fit	6.17	0.92												–	−0.42*
13	Picture complexity	3.23	1.16													–

For all scales, higher scores are indicative of higher ratings. *p < 0.01.

Pictures representing BOI-vocabulary words were chosen from the International Picture Naming Project (IPNP, Bates et al., 2003), if available (69 items). The IPNP is a database containing 795 picture stimuli of objects and actions, providing naming norms for seven different languages, including German. The pictures are simple black-and-white line drawings. Pictures not found in the database of the IPNP (total of 54 words) were selected by the first author, to match the style of the existing pictures as closely as possible (see Appendix B for examples). To ensure that effects found could not be attributed to the complexity of the chosen picture or the fit of the picture to the word, we collected ratings for these variables from ten new participants, who were doctoral and postdoctoral employees of the department working in a different field to the current study. On a 7-point Likert scale, participants rated each picture, based on the questions “how complex is the picture” (picture complexity) and “how well does the picture fit the word” (picture fit). Results showed an excellent interrater reliability amongst the ten raters, Cronbach’s alpha = .93, ICC = .93 for picture fit and Cronbach’s alpha = 0.92, ICC = 0.92 for picture complexity.

For the actual test, children were positioned in front of a computer screen, on which two pictures were shown, the target picture and a distractor. The distractor item was randomly drawn from the other 122 items. Each item was therefore presented twice, once as the target word and once as a distractor in a randomized fashion (i.e., left/right). Children were instructed to look at the pictures while simultaneously hearing a pre-recorded word. The orally presented word matched the target picture on the screen. The children were required to identify the corresponding picture by pressing the equivalent buzzer as quickly as possible. Children were asked to return their hands to a resting position next to the buzzer after each trial. To make sure the participants understood the task, three practice trials were held. Here the words were not presented via headphones, instead the experimenter spoke the words to make it possible to intervene immediately should the task be executed falsely, by providing constructive corrective feedback (e.g., re-explaining the task, seldom required after the first practice trial). To ensure a standardized and exact procedure, the experiment was conducted and reaction times were recorded using the software E-Prime 3.0.

***General Vocabulary.*** The Peabody Picture Vocabulary Test (PPVT 4), German Adaptation (Lenhard et al., 2015), was used to assess participants’ receptive vocabulary performance. In this test, the experimenter stated a word (noun, adjective or subject) and participants had to indicate which of the four pictures matched the presented word. The test increased in difficulty and was continued until a ceiling criterion was reached or the test ended. Using the tables provided in the manual, the raw scores were transformed into standardized age-related scores. The test shows excellent internal consistency, Cronbach’s α = 0.97 (Lenhard et al., 2015).

***Reasoning.*** To obtain an intelligence estimate and therefore a general control for IQ, two subtests of the Wechsler Preschool and Primary Scale of Intelligence, German Adaptation (WPPSI-GA), Matrices and General Knowledge, were administered (Petermann and Lipsius, 2014). These two tasks were chosen because they (a) do not require FMS in responding, as is the case with block design, (b) represent one task each from a verbal and spatial domain, (c) are not conceptually close to (BOI-)vocabulary, and (d) are brief to administer. The Matrices subtest required participants to complete partial patterns by selecting the missing part out of five possible answers. The general knowledge subtest assessed participants’ knowledge about events, localities and personalities. Here again, the tests started out easier and increased in difficulty over time. Both tests were continued until a ceiling criterion was reached or the test ended. To obtain a general intelligence estimate, both scores were combined into a single z-factor, with factor loadings of 0.79 each, explaining 62.82% of total variance.

**Ethics Approval Statement.** The study was carried out in accordance with the recommendations of the Ethical Principles of the German Psychological Society (DGP) and approved by the Ethics Committee of the University. Written informed parental consent was obtained and children gave their verbal assent prior to test administration, in accordance with the Declaration of Helsinki.

### Results for Experiment 1

As a result of illness, four children completed one session only, and three participants’ data were excluded because experimental instructions appeared to have been misunderstood, leaving 70 of the original 77 participants. To ensure that only correct responses were included in the analyses, reaction times following an incorrect response were omitted. Additionally, four words (blouse, cliff, needle, zoo) were dropped from subsequent analyses, as error rates for these words were 20% or higher. On an individual level, reaction times were corrected for outliers that lay three standard deviations outside of the individual response means of high, low and medium BOI words. In total 10.03% of responses were omitted.

To create a general estimate of FMS, a factor analysis was conducted, showing, that all FMS tasks (posting coins with the dominant and the non-dominant hand, threading beads, maze tracing) loaded onto a single factor (factor loadings of 0.86, 0.78, 0.90 and 0.58 respectively, while explaining 62.31% of the variance). Therefore, the scores were combined to be represented by a single z-factor (similar to the analysis reported by Suggate and Stoeger, 2014).

Descriptive statistics of age, IQ estimate, general vocabulary, FMS, and BOI reaction latencies as well as accuracy rates are presented in Table 2. Distribution, skewedness and kurtosis were evaluated. With the exception of the BOI response latencies, the distributions showed little skewness or tail-heaviness and appeared to be normally distributed. Regarding the BOI reaction latencies, the data showed a strong leptokurtic distribution, with kurtosis values of 29.34 (Low BOI reaction) and 36.40 (High BOI reaction). When analysing response latencies to high and low BOI words, a significant BOI effect emerged, *t*(69) = 5.01, *p* < 0.001, showing that responses to high BOI words were significantly faster than to low BOI words. Following, gender differences were assessed. Female participants showed overall better scores in the maze tracing task, *t*(71) = 2.08, *p* = 0.04. No further differences were found (all *p*s > 0.11).

**TABLE 2:** Descriptive statistics for preschool sample.

	*M*	*SD*	*n*	*Min*	*Max*
Age (months)	60.40	10.73	77	39.00	79.00
**IQ estimate**					
Matrices	9.44	3.27	75	1.00	23.00
General Knowledge	9.80	3.17	70	1.00	16.00
IQ factor (*Z*-score)	−0.08	1.19	68	−2.60	3.77
General Vocabulary	47.33	9.36	69	27.00	69.00
**FMS**					
Coin-slotting	10.26	4.75	73	1.00	41.00
Bead threading	9.80	2.96	73	4.00	15.00
Maze Tracing	7.12	3.95	73	1.00	14.00
FMS factor (*Z*-score)	0.02	1.02	73	−2.45	3.66
**Response latencies**					
Average reaction latency (ms)	1512.22	775,79	70	116.00	10685.00
High BOI Reaction latency (ms)	1473.52	530.76	70	819.56	5134.31
Low BOI Reaction latency (ms)	1578.72	548.40	70	876.26	5224.65
Correct responses	110.66	17.25	70	56	122

*FMS, fine motor skills; ms, milliseconds.*

**Links between lexical ratings.** To examine the relationship between the word ratings (BOI and body-part), the picture ratings (complexity and fit) and the different word variables (semantic neighbourhood, semantic diversity, length, number of syllables, and frequency class), correlation analyses were conducted and are presented in Table 1. Results showed significant correlations among most lexical ratings and variables, e.g., moderate to strong correlations among BOI rating and hand, *r* = 0.69, *p* < 0.001, as well as arm ratings, *r* = 0.47, *p* < 0.001, emerged.

**FMS and response latencies.** To investigate the role of FMS and body-part association in the processing advantage of high BOI words, analyses using mixed-effects models for experimental research designs (codes were extracted from Hilbert et al., 2019) were conducted. Mixed-effects models can account for nested data and are less susceptible to missing data, violations of normality, and allow for random intercepts and item level analysis. Analyses were conducted using the package *lme4* (Bates et al., 2015) in *R Studio* (RStudio Team, 2016).

As a first step, the unconditional model was calculated to assess the relation of within-subjects to between-subjects variance. An intraclass correlations coefficient (ICC) of.46 was obtained revealing a significant inter-subject variance, therefore supporting the decision to use a mixed-effects model. In a second step, we modelled individual intercepts using random effects but modelled slopes as fixed effects due to model convergence problems. To predict children’s response latencies, FMS, intelligence, age, and general vocabulary were entered as Level-1 covariates, which significantly improved the model, χ^2^(4) = 21.94, *p* < 0.001. BOI ratings, body-part association ratings (hand, arm, foot, and mouth), picture ratings (fit and complexity) and word variables (age of acquisition, semantic neighbourhood, semantic diversity, length (letters), syllables, and frequency class) were then entered as Level 2 covariates (see Table 3 for results and more detail). Adding these variables explained the data significantly better than the first model χ^2^(14) = 397.55, *p* < 0.001. Lastly, we tested for interactions between FMS and the different word rating measures for BOI and body-part association ratings (hand, arm, foot, mouth), which again improved the model significantly, χ^2^(5) = 27.88, *p* < 0.001.

**TABLE 3:** Mixed effects model predicting childrens’ response latencies from fine motor skills (FMS), cognitive factors, age in months, word variables, BOI ratings, and body-part association ratings.

	Predictor	Estimate	*SE*	*df*	*t*
Response latency (in ms): *R*^2^_marginal_ = 0.154; *R*^2^_conditional_ = 0.513					
**Fixed parts**					
Level 1	(Intercept)	4058.47	606.02	69.27	6.70***
	FMS	−1.95	66.64	85.82	−0.03
	Intelligence	10.44	63.91	64.71	0.16
	General vocabulary	−11.50	7.75	64.74	−1.46
	Age	−28.63	6.11	64.79	−4.69***
Level 2	Age of acquisition	38.21	6.42	7316.03	5.95***
	Semantic neighbourhood	−0.93	0.42	7316.09	−2.21*
	Semantic diversity	145.47	36.22	7315.99	4.02***
	Length (letters)	6.85	4.97	7316.03	1.38
	Syllables	−23.07	15.35	7315.94	−1.50
	Frequency class	10.89	4.24	7315.95	2.57*
	Picture complexity	11.43	6.31	7315.92	1.81
	Picture fit	−115.38	9.03	7315.94	−12.78***
	BOI rating	−2.00	5.90	7316.04	−0.34
	Hand rating	−11.46	8.32	7316.01	−1.38
	Arm rating	10.37	10.05	7315.94	1.03
	Foot rating	−3.79	4.86	7316.09	−0.78
	Mouth rating	−8.22	4.19	7316.09	−1.95
Interactions	BOI rating X FMS	8.40	4.91	7316.19	1.71
	Hand rating X FMS	−17.92	8.45	7316.61	−2.12*
	Arm rating X FMS	−13.24	10.33	7316.40	−1.28
	Foot rating X FMS	19.61	4.88	7316.31	4.02***
	Mouth rating X FMS	−4.56	4.10	7316.49	−1.11
**Random parts**					
	Intercept variance	232308			
	Residual variance	314707			
	Observations	7404			
	*N*_subjects_	70			

Ms, milliseconds. *p < 0.05. **p < 0.01. ***p < 0.001.

The model in Table 3 addresses the main hypothesis that children’s FMS predict the response latencies for BOI-vocabulary. In Table 3, there is a strong interaction between FMS and hand rating, indicating that a higher hand rating as well as better FMS lead to faster response latencies. Furthermore, an interaction between FMS and the foot rating emerged, indicating that a higher rating for the body-part foot and lower scores in the FMS tasks results in slower reaction times. The model further shows that FMS alone did not have a significant influence. A similar pattern was found for the variables intelligence and general vocabulary, whereas the age of the participants significantly predicted response latencies. Controlling for lexical variables was also a crucial addition to the model. A higher age of acquisition, a less dense semantic neighbourhood, a greater degree of semantic diversity as well as a higher frequency class (meaning less frequently occurring) all lead to slower reaction times. Including the picture fit in the model also proved to have an influence on reaction time latencies, as words with a better picture fit elicited a significantly faster reaction.

### Discussion for Experiment 1

We attempted to replicate, clarify, and extend previous work examining links between BOI (Suggate and Stoeger, 2014, 2017; Lund et al., 2019), FMS, and body-part associations, using a tighter experimental method and a range of controls for IQ, general vocabulary, and lexical and stimulus features. First, our results add to the work finding shorter response latencies for high-BOI words in children (Inkster et al., 2016; Suggate and Stoeger, 2017). Second, we did not find a significant link between participants FMS and reaction times to high BOI words, which is contrary to earlier studies (e.g., Suggate and Stoeger, 2014, 2017). However, we discovered a special role for words associated with the body-part hand. More precisely, participants with greater FMS responded more quickly to words that have a strong association with the hand. Furthermore, we found evidence that children with lower FMS reacted less rapidly to words associated with the body-part foot.

Interestingly, a study by James and Maouene (2009) showed that verbs connected to a hand versus foot movement activated different regions of the frontal cortex. Given the strong connection between the hand and FMS, it seems reasonable for words associated with the hand to show a positive link with FMS, while words associated with the body-part foot show a negative link. The strong correlation between hand and BOI ratings (*r* = 0.69, see Table 1) may have made the link between BOI and fine motor skills redundant. As previously discussed, BOI involves the whole body, including interactions with the hand and the foot. Therefore, the interaction between BOI and FMS might have been suppressed in our study by the contrary findings for the body-parts hand (facilitatory effect) and foot (inhibitory effect). As to why previous studies have found links between BOI and FMS, one could hypothesize that the selection of stimuli could be the deciding factor. We opted to assess different body-part-associations and their link to FMS, thus choosing words strongly associated with just one body-part whereas previous studies did not include body-part associations and BOI in a single study, nor were words selected to be particularly associated with body-parts. Conceivably, vocabulary development is initially, as found in previous research (Maouene et al., 2008), linked to body-parts, with this branching into abstract and general BOI representations over the course of development. However, to determine whether body-part associations are specific to childhood, or generalize to an adult population, a further study is needed.

## Experiment 2

Previous studies on the role of BOI for the link between FMS and vocabulary development (Suggate and Stoeger, 2014, 2017) have solely focused on preschool children. We thus sought out to assess whether these findings extend to an adult population. Furthermore, the inclusion of this adult sample seemed highly promising, as it may help us gain insight into the underlying mechanisms. Specifically, as children in Experiment 1 showed a distinct processing advantage for words associated with the body-parts hand but not for words with a high BOI, several possibilities arise. On the one hand, these effects may disappear in adults, thus supporting a functionalism account (Iverson, 2010), as FMS should play a substantial role during childhood language learning but not during adulthood. On the other hand, the results may extend to an adult population, which in turn may be interpreted more along the line of the shared activation account (e.g., Anderson, 2007; Penner-Wilger and Anderson, 2013).

### Materials and Method for Experiment 2

**Participants.** A total of 80 adults aged 19–30 (*M* = 23.60; *SD* = 2.42) of which 66.25% were female took part in this study. 95% of the participants were right-handed, 2.50% left-handed and 2.50% ambidextrous. All were native speakers of German. The participants gave written informed consent and received 10 € as compensation. The study was approved by the University Ethics Committee.

**Measures and procedure.** Students’ demographic information concerning age, marital status, highest school qualification, job training and current job was gathered via a questionnaire.

As in Experiment 1 the M-ABC 2 was used to assess FMS. However, the tasks vary depending on the age group. Hand dominance was determined simply by asking the adults as to whether they were left or right handed (or ambidextrous). For the adult population until age 16, the FMS tasks included turning pegs with the dominant and the non-dominant hand, building a triangle with nuts and bolts and maze tracing. Although the Movement-ABC is only normed for individuals up to age 16, we reasoned that FMS development is thought to plateau around this age and hence, to also preserve task continuity with study 1, we opted for this test battery. Again, two trials were possible for each task, depending on whether the first trial was completed within a certain time frame (pegs and triangle) or completed without a mistake (tracing).

The same BOI-vocabulary test and general vocabulary test (PPVT-4) were used as in Experiment 1. To obtain an intelligence estimate, two subtests of the Wechsler Adult Intelligence Scale, German Adaptation (WAIS-GA), Matrices and General Knowledge, were used, equivalent to the WPSSI in study one (Petermann and Petermann, 2014). Both scores were combined into a single z-factor, with factor loadings of.74 each, explaining 55.23% of total variance. The experimental procedure was almost identical to study one, except that due to adults’ greater attention spans, the experiment was conducted in one session of approximately sixty minutes. Undergraduate students in primary school education were trained by the first author to conduct the experiment.

### Results for Experiment 2

One participant performed the peg task incorrectly and was eliminated from the analyses, leaving 79 out of 80 participants. In accordance with experiment 1, only correct responses were admitted to the analysis, while incorrect responses were omitted. No words were dropped from the subsequent analyses, as error rates were all below 10%. On an individual level, reaction times were corrected for outliers that lay 3 standard deviations outside of the individual response means of high, low and medium BOI words. In total, 3.52% of responses were omitted.

As the norms of the M-ABC 2 only extend to the age of sixteen, adults’ M-ABC 2 scores were not converted into standard scores. Instead, the raw scores were used in the analyses. To facilitate interpretation, adult motor scores were converted by multiplying -1, such that greater FMS scores indicate more pronounced FMS levels. Having determined that students’ FMS (peg turning with the dominant and the non-dominant hand, building triangle, maze tracing) loaded onto the same factor (factor loadings of 0.74, 0.26, 0.76 and 0.49 respectively, explaining 35.43% of the variance), the FMS data were combined to be represented by a single factor z-score.

Descriptive statistics of age, IQ estimate, general vocabulary, FMS, reaction latencies and response accuracies were calculated (see Table 4). They showed no skewness or tail-heaviness and appeared to be normally distributed. Analysing the reaction time latencies to high and low BOI words, a significant BOI effect emerged, *t*(79) = −11.68, *SE* = 4.11, *p* < 0.001. Responses to high BOI words were significantly faster than to low BOI words. Gender differences were subsequently analysed. Male participants were overall older, *t*(78) = 2.70, *p* = 0.01, female participants were better at turning pegs with their dominant hand, *t*(77) = 1.00, *p* = 0.004. No further differences were found (all *p*s > 0.06).

**TABLE 4:** Descriptive statistics for adult sample.

	*M*	*SD*	*n*	*Min*	*Max*
Age (months)	282.33	29.01	80	239.00	368.00
**IQ estimate**					
Matrices	9.58	2.13	80	5.00	17.00
General knowledge	10.36	2.68	80	5.00	21.00
IQ factor (*Z*-score)	0.06	0.81	80	−2.18	2.51
General vocabulary	65.06	5.66	80	54.00	73.00
**FMS**					
Peg turning dominant hand (s)	18.31	2.80	79	12.00	25.00
Peg turning non-dominant hand (s)	21.68	9.50	80	14.00	42.00
Triangle construction (s)	32.32	7.13	80	20.00	48.00
Maze Tracing	0.13	0.46	80	0.00	3.00
FMS factor (*Z*-score)	0.00	−1.00	79	1.91	−2.59
**Response latencies**					
Average reaction latencies	770.59	184.06	80	288.00	2828.00
High BOI reaction latency (ms)	743.64	94.91	80	581.51	1018.82
Low BOI reaction latency (ms)	791.63	100.58	80	601.59	1125.95
Correct responses	120.23	2.82	80	109	123

*FMS, fine motor skills; ms, milliseconds.*

### FMS and Response Latencies

As in Experiment 1, mixed-effects models for experimental research designs were used to assess the role of FMS and body-part association in the processing advantage of high BOI words. Firstly, to measure the relation of within-subjects to between-subjects variance, the unconditional model was computed, revealing an intraclass correlation coefficient of 0.25. Secondly, we modelled individual intercepts using random effects. The between-subjects variables FMS, IQ estimates, age, and general vocabulary represented the Level-1 covariates. Entering the level 1 variables did not improve the model significantly, χ^2^(4) = 3.44, *p* = 0.49. The linguistic variables (age of acquisition, semantic neighbourhood, semantic diversity, length (letters), syllables, and frequency class), picture ratings (complexity and fit), and word ratings (BOI and body-part) represented Level-2 covariates. Using this model, significantly more variance was explained than in the previous model, χ^2^(14) = 1225.5, *p* < 0.001. As a third step, interactions between FMS and word ratings were entered into the model, further improving the model, χ^2^(5) = 14.01, *p* = 0.02.

The results of the mixed-effects model can be seen in Table 5. Analyses showed a significant interaction between BOI and FMS, indicating that a higher BOI rating as well as better FMS lead to more rapid responses. No further interactions materialized. As the table shows, none of the between-subjects factors, hence FMS, IQ estimates, general vocabulary or age had a significant influence on adult’s response latencies. Furthermore, we assessed the impact of the different word ratings (BOI and body-part association) on the response latencies and found significant influences of the BOI and the body-parts arm, foot and mouth. Participants showed significantly faster response latencies when words were rated as having a high BOI as well as when a word had a high association with the body-parts foot and mouth. However, they reacted considerably more slowly when a word had a high association to the body-part arm. As in experiment 1, the inclusion of lexical variables proved crucial to the model. All lexical variables, with the exception of semantic neighbourhood, emerged as significant predictors of response latencies. More precisely, participants reacted more slowly to longer and less frequent words as well as words with a higher age of acquisition, a more pronounced semantic diversity and fewer syllables.

**TABLE 5:** Mixed effects model predicting adults’ response latencies from fine motor skills, cognitive factors, age in months, word variables, BOI ratings, and body-part association ratings.

	Predictor	Estimate	*SE*	*df*	*t*
Response latency (in ms): *R*^2^_marginal_ = 0.101; *R*^2^_conditional_ = 0.358					
**Fixed parts**					
Level 1	(Intercept)	1015.01	158.01	78.10	6.42***
	FMS	2.08	12.23	115.02	0.17
	Intelligence	0.50	14.66	73.99	0.03
	General vocabulary	−2.66	2.26	73.98	−1.18
	Age	−0.04	0.40	73.98	−0.10
Level 2	Age of acquisition	9.14	1.53	9276.22	5.97***
	Semantic neighbourhood	−0.07	0.10	9276.08	−0.65
	Semantic diversity	45.44	8.49	9276.10	5.35***
	Length (letters)	8.03	1.17	9276.08	6.85***
	Syllables	−23.41	3.66	9276.09	−6.41***
	Frequency class	8.28	1.00	9276.11	8.26***
	Picture complexity	5.05	1.51	9276.10	3.35***
	Picture fit	−40.48	2.10	9276.11	−19.32***
	BOI rating	−5.26	1.38	9276.11	−3.82***
	Hand rating	−2.15	1.90	9276.10	−1.13
	Arm rating	8.40	2.36	9276.13	3.56***
	Foot rating	−11.87	1.14	9276.06	−10.39***
	Mouth rating	−2.27	1.00	9276.09	−2.27*
Interactions	BOI rating X FMS	−2.65	1.10	9276.12	−2.41*
	Hand rating X FMS	−0.82	1.85	9276.12	−0.44
	Arm rating X FMS	0.94	2.31	9276.21	0.41
	Foot rating X FMS	−1.24	1.11	9276.08	−1.12
	Mouth rating X FMS	0.36	0.92	9276.10	0.39
**Random parts**					
	Intercept variance	8877			
	Residual variance	22209			
	Observations	9373			
	*N*_subjects_	79			

ms, milliseconds. *p < 0.05. **p < 0.01. ***p < 0.001.

### Discussion for Experiment 2

Our goal in Experiment 2 was to expand the understanding of how FMS relate to lexical processing, with a particular focus on the role of BOI and body-part associations in an adult population. The most important finding is the significant interaction between BOI rating and FMS, as well as the lack of interaction between any body-part association and FMS. Participants with greater FMS reacted more rapidly to words with a high BOI, whereas body-part association played no significant role. This is contrary to the results found in experiment 1, where a unique role for the hand was found. It appears as though the link between FMS and vocabulary in children may be specific to words containing a high association to the body-part hand, whereas in adults, this link seems to incorporate words that have a high BOI rating, thus extending previous work (Suggate and Stoeger, 2014, 2017).

Additionally, the results of the study show a significant facilitatory effect for high BOI words over low BOI words. This is consistent with findings from other studies assessing the BOI effect with adult populations (e.g., Siakaluk et al., 2008a,b). Furthermore, influences of body-part associations were found. Participants showed more rapid responses to words associated with the body-parts foot and mouth and slower responses to words, with a strong association to the body-part arm. Moreover, whereas all word variables, with the exception of semantic neighbourhood, emerged as significant predictors for the adult population in Experiment 2, only age of acquisition, semantic neighbourhood, semantic diversity and frequency class, appeared to have an influence on lexical processing in children (Experiment 1). Finally, the factor analyses for combining the Movement-ABC measures into a single FMS score explained less variance than in the child sample in study 1. Given that this test battery is only developed as a screening and normed up until age 16, this raises concerns that this measure may be less suited to capturing the upper performance range, perhaps compromising the current findings. Therefore, in the next study we incorporated a pegboard task designed to capture more variance while replicating study 2.

## Experiment 3

In study 2, participants performed a FMS task that was developed as a screening for children and adolescents, giving rise to the possibility that it did not have a sufficiently high ceiling to capture adults’ true performance. Accordingly, we replicated study 2, but replaced the Movement ABC FMS tasks with a classic pegboard task commonly used to assess FMS in adults (e.g., Gallus and Mathiowetz, 2003). Although the Movement ABC task in Study 2 contained a pegboard task, this had only 12 pegs and a limited number of trials, versus the 24 pegs across four trials in the current study. We hypothesized that FMS would predict response latencies, as would BOI ratings and the interaction between the two. We expected similar contributions of the body-parts as found in Study 2.

### Materials and Method for Experiment 3

Participants were 71 adults, 62% female, aged 23.85 years (*SD* = 3.50) and 88.7% indicated that they were right-handers. The participants gave written informed consent and received no compensation. Participants were tested individually in a single session by trained education students as part of their research theses. The methods in study 3 followed those of study 2 with the following exceptions. First, given that vocabulary and the intelligence tasks did not uniquely predict response latencies in study 2, these were dropped.

Additionally, instead of using the Movement ABC, we used a Pegboard task. The pegboard task contained four trials in which participants had to first insert 24 small metal pegs (3mm diameter, 3cm long) 5mm holes spaced 1cm apart and in two parallel rows of 12 holes, with the one row being 4cm from the other. The pegs were located in a small container fastened to the end of the pegboard which they held onto with their non-operative hand while they inserted the pegs into the first row, one at a time, as quickly as possible. In the second trial, the participants shifted the pegs from the one row to the next. Both trials were firstly conducted with the right hand, then the left hand. Scores represent the time (s) taken to insert all pegs across all four trials. Internal consistency across the four trials was acceptable, α_cr_ = 0.63.

### Results and Discussion for Experiment 3

Across the four trials, participants required 133.97 (*SD* = 13.17) seconds for the pegboard tasks and complete the BOI task with a 92% accuracy and a mean response latency of 872.41 (*SD* = 137.28) milliseconds. An identical analysis to that presented in Table 5 was conducted, with the exception that an IQ and vocabulary estimate was missing, and the non-significant body part X FMS interactions were trimmed. Response latencies lying outside of three standard deviations of an individual’s mean were removed.

The resulting model was similar to that in study 2, with a host of significant level 2 (lexical) predictors, ICC = 0.11, marginal *R*^2^ for entire model = 0.15. Of particular relevance to the current hypothesis, ratings for BOI, β = −87.73 ms, *p* < 0.001, foot, β = −15.05 ms, *p* < 0.05, and mouth, β = −12.05 ms, *p* < 0.05, were significant predictors, whereas arm, β = −2.06, *p* = 0.86 and hand, β = 12.34, *p* < 0.25, were not. Word frequency (*p* < 0.05) and picture fit (*p* < 0.001) were also significant predictors. As with studies 1 and 2, pegboard scores were multiplied by −1, such that greater scores indicate greater performance. Participant age (*p* = 0.08) was not significant but FMS were, β = 3.13 ms, *p* < 0.05, as was the interaction between FMS and BOI rating, β = −0.57, *p* = 0.001.

Thus, consistent with study 1 and 2, FMS played a significant role in predicting response latencies, as did BOI and body-part association ratings. Beginning with FMS, in study 3 this was a significant predictor of response latencies, whereby adults with greater FMS performed more slowly on the lexical decision task. Words with a higher BOI rating were processed more quickly and there was a positive significant interaction between FMS and BOI ratings, such that greater FMS resulted in more rapid processing of higher BOI words. Accordingly, the data support the hypothesis that FMS create processing advantages for high-BOI words. The finding that foot and mouth were significant and hand and arm were not, probably indicates that body-part is a construct that is closely related to BOI. Perhaps mouth and foot captured aspects of embodiment that were not captured entirely by the BOI ratings, whereas arm and hand words were made redundant by BOI rating and the FMS measures. The current study extends study 2, confirming a link between BOI and FMS using a task that can be expected to capture variance in FMS in adults.

## General Discussion

In three experiments, we examined whether lexical items contain a unique link to the body, with a particular focus on the sensorimotor, or more specifically, fine motor system. A vast literature finds that concepts (Glenberg and Gallese, 2012; Pexman, 2019), including abstract concepts to some extent (Kiefer and Harpaintner, 2020), have conceptual and neurobehavioral roots in peripheral sensorimotor experiences (Heard et al., 2018). Little research has examined to date, whether sensorimotor *performance* relates to lexical processing. Such a line of work would appear to have a number of benefits, providing an additional line of enquiry into the nature of embodiment that is less dependent on imaging studies, which can only infer motor involvement via motor cortex activation. Perhaps most importantly, examining the performance of the fine motor system in relation to lexical processing asks the unique question of whether skill, or nimbleness, in two seemingly unrelated domains is actually interdependent. In this spirit, we conducted the three experiments reported here.

Specifically, although there is some literature on the link between BOI and FMS in children (Suggate and Stoeger, 2014, 2017; Lund et al., 2019), it is, to our knowledge, the first study to examine this in an adult population and extend this to comparatively test the role of body-parts. The first key finding was that results from all three studies showed that performance in FMS was positively related to lexical processing, for words that were embodied (either high-BOI in adults or hand words in children). The second key finding was the differential link between FMS and lexical processing as a function of sample age. Participants in the child population (aged 3 to 6) showed a strong positive link between FMS and words associated with the hand as well as a strong negative relation between FMS and words associated with the foot. The link between BOI and FMS, however, was not significant in this age group. In contrast, we found a strong connection between FMS and high BOI words in the adult population in study 2 and 3.

Our results emphasize the importance of considering BOI as well as body-part associations when assessing the relationship between FMS and vocabulary. Considering the mixed and inconclusive results on the link between FMS and vocabulary (e.g., Wassenberg et al., 2005; Alcock and Krawczyk, 2010; Pagani et al., 2010; Cameron et al., 2012), our results may offer an explanation. It is possible that the degree of embodiment as well as body-part association in previous studies varied in the selected lexical items, leading to partly conflicting results. Future studies should hence attempt to include BOI and body-part association in their study designs to better understand how vocabulary relates to sensorimotor development.

Further, the above findings offer helpful insights into the theoretical mechanisms. As found in previous studies (e.g., Cameron et al., 2012; Suggate and Stoeger, 2014, 2017), age and cognitive factors explained some but not all of the variance between FMS and lexical processing, therefore ruling out maturation or a general cognitive factor as the sole driving force. Consequently, consideration can turn to the functionalism and shared-activation accounts. Children’s FMS and their response latencies showed a positive relation to words associated with the hand and a negative link to stimuli linked to the body-part foot, whereas BOI did not seem to play a role in their lexical processing. One might speculate that this speaks for a functionalistic viewpoint, as children use their hands to interact with things in their environment, therefore facilitating FMS and learning (Iverson, 2010). This concept, however, seems ill-equipped to explain the link found in adults, as developmental processes concerning FMS and language acquisition should be completed by adulthood. Nevertheless, adults’ FMS predict response latencies to BOI words.

Given the persistence of links into adulthood, explaining the current findings from a purely functionalistic viewpoint therefore, seems to be insufficient. Although children primarily show strong links to words associated with the body-part hand, BOI should not be taken out of the picture entirely. Our findings of links between FMS and embodied lexical items (either BOI or body-part association) lead to the assumption that more advanced FMS may result in better lexical processing skills for all embodied lexical items, not just words associated with the body part hand. The results of the adult population further point in this direction, with response latencies for high-BOI words benefitting from greater FMS, over and above the control variables, as postulated by the nimble-hands, nimble-minds hypothesis (Suggate and Stoeger, 2014, 2017). Therefore, tentative evidence that lexical processing partially involves FMS motor representations has been found (Penner-Wilger and Anderson, 2013).

In terms of purely lexical processing without considering the role of FMS, the pattern of findings, from a specific concept (e.g., body-parts) in childhood to less defined concepts (e.g., BOI) in adulthood, correspond to results obtained by Maouene et al. (2008). Consequently, it seems plausible, that children’s response latencies may be influenced more strongly by specific body-part associations, whereas adults’ response latencies show a stronger connection to the broader concept of BOI. Thus, it seems possible that the link between FMS and lexical processing undergoes a shift in underlying mechanisms.

Nevertheless, due to methodological and conceptual reasons, we interpret these findings with caution. Firstly, we assessed reaction times using a manual design (e.g., pressing buzzers) while simultaneously evaluating hand associations. One might argue that pressing a buzzer using the hands enhances the semantic processing of words associated with the hand. A study by Connell and Lynott (2014) shows that directing attention to a specific modality can lead to an enhanced processing of that modality. Then again, a study conducted by Wellsby et al. (2011) showed no difference in the BOI effect between manual and non-manual designs. Furthermore, Buccino et al. (2005) found that reaction times of participants were slower when responding with the hand while listening to hand-action-related sentences, compared to when the response was given with the foot. Accordingly, future studies could assess reaction times using a supplementary control where participants are not forced to use their hands, arms or fingers but rather use looking times.

Secondly, we did not assess environmental motor influences such as children’s actual physical interactions with the objects used as stimuli in the study. Therefore, it is not possible to draw any firm conclusions about the extent to which functionalism may or may not play a role in the link between FMS and lexical processing. We furthermore did not conduct an imaging study enabling us to assess neurological structures underlying cognitive representations. This makes it difficult to directly investigate the neurological basis of the shared-activation account.

Thirdly, all ratings on BOI and body-part associations were acquired from adults. Generalizing these ratings to an early childhood population may prove difficult. Words that adults deem as having a low BOI may be categorized differently by children. Perhaps future work could gather these from children or from parents of young children. For instance the word “star” may be seen as a celestial body by adults (i.e., low BOI), whereas children may associate the word “star” with a star-shaped toy (i.e., high BOI). Furthermore, BOI measures the actual experience one has had interacting with the words’ referent. This experience may differ substantially between adults and children (Inkster et al., 2016).

Finally, we included various linguistic variables (such as age of acquisition, semantic neighbourhood, etc.) that have been shown to influence semantic processing (e.g., Buchanan et al., 2001; Balota et al., 2004; Cortese and Khanna, 2007). Nevertheless, there may be other lexical variables that drive the link between FMS and lexical processing and may only be masked by the BOI or the body-part association.

The current findings have both clinical and educational implications. From a clinical viewpoint, it is interesting to view our findings in light of object exploration. A number of researchers have found compelling evidence linking object exploration and communicative and language development in typically developing children (Ruddy and Bornstein, 1982), autistic children (Hellendorn et al., 2015), children with developmental delays (Hellendorn et al., 2015) and preterm infants (Zuccarini et al., 2018). The findings underline the functionalistic viewpoint in children, as very early object interaction seems to be essential for conceptual and linguistic knowledge. A longitudinal approach assessing early object exploration and later language skills considering it’s interaction with the BOI or body-part association may lead to further interesting insights in the underlying passageways.

Concerning the educational implications, our findings highlight the notion that language development does not occur independently from motor development. From antiquity to the 20th century, psychologist, philosophers and educationalists, such as Jean Piaget and Maria Montessori, have attributed an important role to the sensorimotor system in semantic processing and mental development (e.g., Piaget, 1983; Montessori, 2013). Furthermore, studies have shown an influence of motor training on cognitive variables (e.g., McCormick and Schnobrich, 1971; Westendorp et al., 2014). Our findings once more underline a motor basis for language processing (Glenberg and Kaschak, 2002). This suggests that children should be given opportunities to engage in (fine) motor actions involving the hands, which in turn could facilitate lexical development. Future studies may want to focus on motor knowledge and its link to language and motor skill development. A promising approach may be via intervention studies, as these allow controlling for motor experience, providing causal data on the relations between FMS and vocabulary development.

## Data Availability Statement

The raw data supporting the conclusions of this article will be made available by the authors, without undue reservation.

## Ethics Statement

The studies involving human participants were reviewed and approved by Ethikkommission der Universität Regensburg. Written informed consent to participate in this study was provided by the participants’ legal guardian/next of kin.

## Author Contributions

RW, HS, and SS conceived and planned the experiments. RW carried out the experiments 1 and 2, wrote the first draft of the manuscript. SS conducted experiment 3, wrote the section on study 3 of the manuscript. RW and SS performed the statistical analysis. All authors provided critical feedback and helped shape the research, analysis and manuscript.

## Conflict of Interest

The authors declare that the research was conducted in the absence of any commercial or financial relationships that could be construed as a potential conflict of interest.

## Footnotes

1 http://wortschatz.uni-leipzig.de/de
