# Supplementary material for: Fine Motor Skills and Lexical Processing in Children and Adults
Source: Front Psychol. 2021 May 12;12:666200. doi: 10.3389/fpsyg.2021.666200 (PMC8149613; doi:10.3389/fpsyg.2021.666200)
Supplement: Supplementary file 1 [file Data_Sheet_1.docx]

**Appendix A. Items used in the experiment**

| Item | Word (English translation) | Item | Word (English translation) | Item | Word (English translation) |
| --- | --- | --- | --- | --- | --- |
| 1 | Apfel (apple) | 42 | Handschuh (glove) | 83 | Ritter (knight) |
| 2 | Ärmel (sleeve) | 43 | Handtasche (purse) | 84 | Rock (skirt) |
| 3 | Auto (car) | 44 | Hemd (shirt) | 85 | Schiff (ship) |
| 4 | Axt (axe) | 45 | Herz (heart) | 86 | Schreibtisch (desk) |
| 5 | Bach (creek) | 46 | Hexe (witch) | 87 | Schuh (shoe) |
| 6 | Ball (ball) | 47 | Himmel (sky) | 88 | Schüssel (bowl) |
| 7 | Bank (bench) | 48 | Hocker (stool) | 89 | Schwamm (sponge) |
| 8 | Beamter (clerk) | 49 | Hose (pants) | 90 | Schwert (sword) |
| 9 | Besen (broom) | 50 | Hund (dog) | 91 | Seife (soap) |
| 10 | Bett (bed) | 51 | Hut (hat) | 92 | Seil (rope) |
| 11 | Blatt (leaf) | 52 | Jacke (coat) | 93 | Sitz (seat) |
| 12 | ^a^Bluse (blouse) | 53 | Kamm (comb) | 94 | Sofa (couch) |
| 13 | Blut (blood) | 54 | Kette (chain) | 95 | Sonne (sun) |
| 14 | Brille (glasses) | 55 | Kiste (crate) | 96 | Spiel (game) |
| 15 | Brot (bread) | 56 | Kleidung (clothes) | 97 | Spielzeug (toy) |
| 16 | Brunnen (well) | 57 | ^a^Klippe (cliff) | 98 | Stern (star) |
| 17 | Buch (book) | 58 | König (king) | 99 | Stiefel (boot) |
| 18 | Clown (clown) | 59 | Kuchen (cake) | 100 | Stift (pen) |
| 19 | Dach (roof) | 60 | Kuss (kiss) | 101 | Stock (stick) |
| 20 | Dienstmädchen (maid) | 61 | Lächeln (smile) | 102 | Strand (beach) |
| 21 | Drachen (kite) | 62 | Lied (song) | 103 | Strohhalm (straw) |
| 22 | Fackel (torch) | 63 | Lippenstift (lipstick) | 104 | Sturm (storm) |
| 23 | Faden (string) | 64 | Löffel (spoon) | 105 | Tasse (mug) |
| 24 | Fahrrad (bike) | 65 | Messer (knife) | 106 | Tee (tea) |
| 25 | Faust (fist) | 66 | Mond (moon) | 107 | Teich (pond) |
| 26 | Flamme (flame) | 67 | Münze (coin) | 108 | Telefon (telephone) |
| 27 | Flasche (bottle) | 68 | Nacht (night) | 109 | Teppich (rug) |
| 28 | Flöte (flute) | 69 | ^a^Nadel (needle) | 110 | Tor (goal) |
| 29 | Gabel (fork) | 70 | Nagel (nail) | 111 | Traube (grape) |
| 30 | Gefängnis (jail) | 71 | Nest (nest) | 112 | Trommel (drum) |
| 31 | Gehstock (cane) | 72 | Nuss (nut) | 113 | Vase (vase) |
| 32 | Geist (ghost) | 73 | Pedal (pedal) | 114 | Wasserfall (waterfall) |
| 33 | Geschäft (shop) | 74 | Pfad (trail) | 115 | Welt (world) |
| 34 | Geschenk (gift) | 75 | Pistole (gun) | 116 | Werkzeug (tool) |
| 35 | Glas (glass) | 76 | Prinz (prince) | 117 | Wolke (cloud) |
| 36 | Glühbirne (bulb) | 77 | Pumpe (pump) | 118 | Würfel (cube) |
| 37 | Gott (god) | 78 | Puppe (doll) | 119 | Zahn (tooth) |
| 38 | Grab (tomb) | 79 | Rad (wheel) | 120 | Zahnbürste (toothbrush) |
| 39 | Gürtel (belt) | 80 | Rauch (smoke) | 121 | ^a^Zoo (zoo) |
| 40 | Halle (hall) | 81 | Regal (shelf) | 122 | Zug (train) |
| 41 | Hammer (hammer) | 82 | Ring (ring) | 123 | Zunge (tongue) |

*Note.*

^a^ Word excluded in the statistical analyses of experiment 1 as error rate was 20% or higher.

**Appendix B. Examples of the pictures used in BOI Vocabulary Test**

| 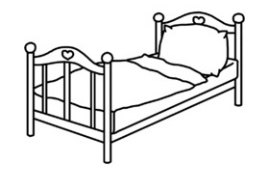 | 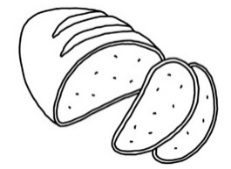 | 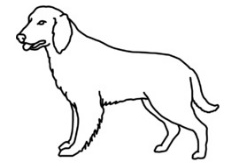 | 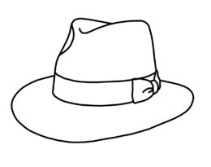 |
| --- | --- | --- | --- |
| Bett (bed) | Brot (bread) | Hund (dog) | Hut (hat) |
| 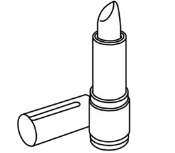 | 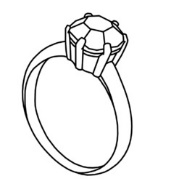 | 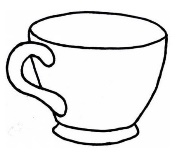 | 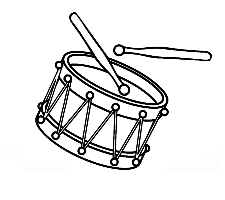 |
| Lippenstift (lipstick) | Ring (ring) | Tasse (mug) | Trommel (drum) |
